# Disparities in Blood Pressure Measurement

**DOI:** 10.1007/s11906-026-01391-6

**Published:** 2026-09-18

**Authors:** Ayan Ali, Liann Abu Salman, Jordana B. Cohen

**Affiliations:** 1 Cardiology Division, Department of Medicine, Feinberg School of Medicine, Northwestern University, Chicago, IL USA; 2 Renal-Electrolyte and Hypertension Division, Perelman School of Medicine, University of Pennsylvania, Philadelphia, PA USA; 3 Department of Biostatistics, Epidemiology, and Informatics, Perelman School of Medicine, University of Pennsylvania, Philadelphia, PA USA

**Keywords:** Equity, Disparities, Blood pressure, Blood pressure measurement, Hypertension, Validation

## Abstract

**Purpose of review:**

We aimed to examine how inaccuracies and inequities in blood pressure (BP) measurement across office, ambulatory, home, and emerging cuffless modalities contribute to disparities in hypertension diagnosis and control, and to identify strategies to improve measurement equity.

**Recent findings:**

Disparities arise from nonstandardized office technique, inappropriate cuff sizing, limited access to validated home and ambulatory devices, reimbursement and implementation barriers, and underrecognition of masked and nocturnal hypertension in populations at higher risk. Cuffless technologies may expand access and reduce cuff-related barriers, but current devices have important limitations in validation, calibration, real-world accuracy, and performance across diverse populations.

**Summary:**

Improving equity requires standardized measurement, access to appropriately sized validated devices and out-of-office monitoring, inclusive device validation, and reimbursement and implementation strategies that reach underrepresented and under-resourced communities. Future research should test whether these approaches reduce diagnostic misclassification and improve BP control and cardiovascular outcomes across populations.

## Introduction

Hypertension is the leading risk factor contributing to cardiovascular morbidity and mortality worldwide and affects nearly half of all adults in the United States [1]. Despite advances in pharmacotherapies as well as emerging interventional approaches for the management of hypertension, adequate blood pressure (BP) control remains a major public health challenge [2, 3]. In the United States, only one in five adults have controlled hypertension, with control rates significantly lower among younger adults, men, and certain racial groups [4]. The disproportionate burden of hypertension reflects longstanding structural inequities that have sustained racial and ethnic disparities in hypertension prevalence and control for over five decades [5, 6]. Black adults continue to have the highest rates of hypertension, are more likely to develop the disease at younger ages, experience greater disease severity and complications such as stroke, heart failure, and chronic kidney disease [7, 8]. These disparities have been extensively reported and are not adequately explained by genetics or biology alone, reflecting the cumulative impact of structural factors, social determinants of health, and inequities embedded within healthcare systems, including those arising from how BP is measured [9, 10]. Inequities in BP measurement may also arise from geographic and socioeconomic barriers, language and accessibility needs, and population-specific challenges with conventional measurement.

Given that accurate BP measurement is fundamental to the diagnosis, monitoring, and treatment of hypertension, addressing measurement related inequities is essential to improving outcomes and advancing equity in hypertension [1]. Measurement inaccuracies can undermine hypertension diagnosis and control and may compound existing disparities in cardiovascular outcomes. Moreover, reliance on office-based measurements in clinical practice fails to capture the full spectrum of hypertension phenotypes, some of which carry substantial cardiovascular risk and occur more frequently in certain populations. [8, 10–12]. This review examines the current landscape of disparities in BP measurement, with a focus on office-based, ambulatory, home, and emerging modalities that may contribute to inequities in BP control Table 1. Through this lens, we explore system-level strategies, technological innovations, and patient-centered interventions aimed at improving the accuracy and equity of BP assessment.

**Table 1:** Inequities across blood pressure measurement modalities

Modality	Key Barriers to Equitable Measurement	Potential Consequences	Priorities for Equitable Measurement
Office-based BP Monitoring	• Cuff sizing • Nonstandardized technique • Limited repeated measures	• Systematic measurement error and misclassification • Missed out-of-office hypertension	***For health systems:*** • Implement standardized automated BP measurement protocols including staff training and retraining • Maintain a full range of cuff sizes • Establish pathways for appropriate out-of-office confirmation
Ambulatory BP Monitoring	• Limited availability and infrastructure • Limited reimbursement • Poor tolerability	• Inequitable access to detection of masked and nocturnal hypertension	***For payers:*** • Expand coverage for performance and interpretation • Expand coverage to additional clinically appropriate indications ***For health systems:*** • Develop sustainable workflows that support device placement, return, and interpretation
Home BP Monitoring	• Device cost and validation • Cuff size availability • Patient training • Digital burden	• Inequitable access to reliable longitudinal BP assessment	***For payers:*** • Cover validated devices and appropriately sized cuffs • Provide better coverage for patient training and data interpretation ***For health systems:*** • Pair self-monitoring with standardized patient training and clinical support
Cuffless BP Monitoring	• Calibration • Sensor performance • Skin tone and other physiologic factors that may affect device accuracy • Algorithm development and validation	• Potential technology-related measurement bias	***For regulators and standards bodies:*** • Require technology-specific validation across diverse populations • Require technology-specific clinical validation across intended-use conditions

## Office-Based Blood Pressure Measurement

Office-based BP measurement (OBPM) is the most commonly used method for diagnosing and monitoring hypertension. It is performed either with a validated, fully automated oscillometric device or manually via auscultation of the brachial artery with a stethoscope [1]. Numerous sources of error in OBPM complicate the accurate diagnosis and effective management of hypertension. These include the technical and cognitive competencies necessary for auscultatory readings, appropriate patient positioning, selection of correctly sized cuffs, adherence to standardized pre-measurement conditions, use of validated automated devices and repeated measurements to improve reliability [1, 13, 14]. The accuracy of measurement is heavily dependent on correct techniques and standardized procedures that are often lacking in clinical practice.

Several important sources of error in OBPM may be unevenly distributed across populations. The most common office-based error is using inappropriate cuff size for arm circumference, with under-cuffing large arm circumference accounting for 84% of these errors [1]. In a crossover trial that randomized participants to different cuff sizes, using a regular cuff in participants requiring an extra-large cuff overestimated systolic BP by roughly 20 mmHg [15]. This has important implications as obesity prevalence in patients with hypertension is highest among Non-Hispanic Black adults, followed by Hispanic/Latino adults, with Non-Hispanic White adults and Asian American adults having the lowest obesity prevalence [16]. These challenges point to an opportunity to enhance both the precision and equity of hypertension care by addressing common OBPM errors. Arm circumference alone may also be insufficient to ensure appropriate cuff fit, as individuals with a large arm circumference relative to upper-arm length may be unable to accommodate an appropriately sized cuff without encroachment on the antecubital fossa; population differences in upper-arm anthropometry may further contribute to differential risk of poor cuff fit [17].

Moreover, OBPM fails to capture key hypertension phenotypes that differ in prevalence across racial and ethnic groups. Masked hypertension, defined as normal OBPM with elevated out-of-office BP is very common in Black patients [11]. Data from the Jackson Heart Study, a large population-based cohort found a prevalence of masked hypertension of 25.9% among Black participants with normal OBPM [11, 18]. Nocturnal hypertension, defined as mean asleep BP of ≥ 120/70 mmHg, is another hypertensive phenotype that is independently associated with increased cardiovascular risk [1, 19]. The American Heart Association (AHA) reports that nocturnal hypertension is particularly prevalent in Black adults in the United States, with estimates exceeding 40% in this group, compared to around 20% in White adults [1, 7, 19]. Additionally, non-dipping BP, characterized by less than a 10% decline in BP from daytime to nighttime, is observed in up to 65% of Black adults [1, 3]. These abnormal nighttime BP phenotypes are key contributors to masked hypertension and are not detectable through routine OBPM, leading to underdiagnosis and missed opportunities for intervention in these high-risk patient subgroups. Thus, reliance on OBPM alone can obscure true disease burden and may propagate racial disparities in hypertension outcomes. Improving the consistency of OBPM through standardized protocols, provider training, and integration of validated automated devices is necessary, but insufficient in achieving equitable hypertension without also expanding access to out-of-office BP monitoring.

### Ambulatory Blood Pressure Monitoring

The United States Preventive Services Task Force (USPSTF) and the AHA historically emphasized ambulatory BP monitoring (ABPM) as the gold standard for confirming the diagnosis of hypertension [20–22]. ABPM provides insights into 24-h BP variability, unveiling hypertension phenotypes that OBPM cannot detect such as masked, white coat, and nocturnal hypertension [1, 23]. Conventional OBPM has lower diagnostic performance, with a sensitivity of 51% and specificity of 88% compared to 24-h ABPM, which remains the most sensitive and specific method for diagnosing hypertension [21]. Despite its clinical value, ABPM remains significantly underutilized, and barriers to its implementation may be particularly consequential in resource-limited settings.

Several barriers contribute to underuse of ABPM. First, ABPM is not widely available in primary care settings, as many practices lack the necessary equipment and infrastructure to support its implementation [1, 21]. Barriers in utilization include the upfront cost of acquiring ABPM devices and software, the need for staff training and the development of standardized protocols for device fitting and data interpretation, all of which require substantial initial investment [1, 21, 24]. This is compounded by historically low reimbursement rates from insurance providers for ABPM use, particularly Medicaid, which can impede implementation [1, 21]. Even when reimbursement is available, it is modest and the administrative burden of securing coverage may further hinder more widespread adoption of ABPM. Overall, historical Medicare claims data demonstrate extremely low use, with ABPM claims submitted for approximately 0.1% of beneficiaries [25]. As a result, these barriers may disproportionately affect resource-limited settings and historically marginalized communities, where both provider capacity and patient access to advanced diagnostic tools are more constrained.

In addition to logistical and systemic barriers to implementing ABPM, both patient and provider-related factors contribute to its underutilization [24, 26]. Because ABPM requires 24-h of cuff wear, it may disrupt activities of daily living, work, caregiving responsibilities or social activities. Additionally, there may be discomfort associated with repeated cuff inflation and sleep disturbances during overnight monitoring that can affect patient adherence, particularly compromising the assessment of nocturnal BP patterns [26]. From a provider perspective, limited familiarity with the appropriate indications for ABPM, as well as uncertainty regarding interpretation of the resulting data, may further limit its routine use in clinical practice [21, 24].

### Home Blood Pressure Monitoring

Home BP monitoring (HBPM) is a more accessible alternative to ABPM for many patients and has demonstrated utility in hypertension management [27]. Compared to OBPM, HBPM can better capture white coat and masked hypertension, thereby enhancing detection of high-risk phenotypes and supporting more equitable hypertension outcomes. For this reason, the AHA/American College of Cardiology, European Society of Cardiology, and European Society of Hypertension recommend either ABPM or HBPM for the diagnosis of hypertension in all patients [1, 3, 28–30].

Although HBPM avoids some of the infrastructure and tolerability barriers of ABPM, it has distinct limitations.. Many HBPM devices are not sufficiently validated for accurate BP measurement and require careful selection and patient instruction to ensure appropriate standardized use [10, 31]. Additional barriers to HBPM use include patient out-of-pocket cost and limited availability of properly sized cuffs. Larger cuff sizes, which are essential for accurate readings in patients with larger arm circumferences, are often more difficult to obtain commercially than regular-sized cuffs [32]. Over half (51%) of U.S. adults with hypertension require a large or extra-large cuff, including 57% of non-Hispanic Black adults, and 54% of Hispanic adults, and 51% of non-Hispanic White adults, representing a meaningful population-level difference in cuff-size requirements [33]. Although most contemporary HBPM devices provide wide-range cuffs accommodating arm circumferences up to 42 cm, 12% of Black adults have arm circumferences outside the supplied ranges of popular devices, compared with 7% of White, 5% of Hispanic, and 2% of Asian adults [32]. When an appropriately sized upper-arm cuff cannot be used because of arm size or geometry, a validated wrist BP monitor is a reasonable alternative option [17]. However, accurate wrist BP measurement requires correct positioning over the radial artery with the wrist maintained at heart level, making specific patient education particularly important [34]; these additional technique requirements may pose barriers for individuals with limited health literacy [35]. Furthermore, some devices do not automatically record or transmit readings, requiring patients to manually document results, which can introduce errors and reduce reliability, especially among individuals with lower health literacy or limited digital proficiency [1].

Although barriers to HBPM may themselves contribute to inequities, home-based measurement also has the potential to overcome important barriers inherent to office-based care and ABPM. Because HBPM does not require repeated travel to a healthcare facility or access to specialized ABPM infrastructure, it may be particularly valuable for patients in rural and other resource-limited settings, where longer travel distances and shortages of healthcare professionals can limit access to hypertension diagnosis and follow-up. When coupled with remote clinical support, HBPM can also facilitate longitudinal BP assessment and treatment without repeated in-person visits. Home BP telemonitoring with remote hypertension management has been shown to be feasible in a cohort including rural and low-income patients, although broader implementation remains dependent on access to validated devices, connectivity, digital literacy, and clinical infrastructure [36, 37]. Barriers are even greater for detecting nocturnal hypertension, as most HBPM devices do not measure nighttime BP and validated nocturnal HBPM devices are not widely available or affordable [12, 27]. Thus, ABPM remains the most established approach to assess nighttime BP. Expanding access to validated HBPM and the infrastructure needed to support remote BP management may therefore help address some geographic and healthcare-access disparities, while ABPM remains necessary for phenotypes that cannot be adequately characterized with conventional HBPM.

### Cuffless Blood Pressure Monitoring

Cuffless BP monitoring has the potential to address several barriers inherent to conventional cuff-based measurement [38, 39]. Wearable and mobile devices may obtain intermittent or continuous BP estimates without repeated cuff inflation and could permit frequent measurements during sleep, daily activities, or other settings that are difficult to capture with OBPM, conventional ABPM, or HBPM. By eliminating the need to select and position an appropriately sized cuff, cuffless technologies could also reduce error from miscuffing and expand monitoring options for individuals with very large arm circumferences or other barriers to cuff-based measurement. Their portability may be particularly valuable in rural and under-resourced communities, where travel, device availability, and access to training on appropriate cuff-based measurement can limit out-of-office BP assessment. Cuffless monitoring could also ultimately benefit populations in whom conventional cuff-based measurement itself is particularly challenging. For example, BP assessment in children can be complicated by movement, poor tolerance of repeated cuff inflation, frequent white-coat hypertension, and challenges completing ABPM. Cuffless approaches could potentially mitigate some of these barriers, although rigorous pediatric-specific validation is needed before they can be relied upon clinically [38].

**Fig. 1:**
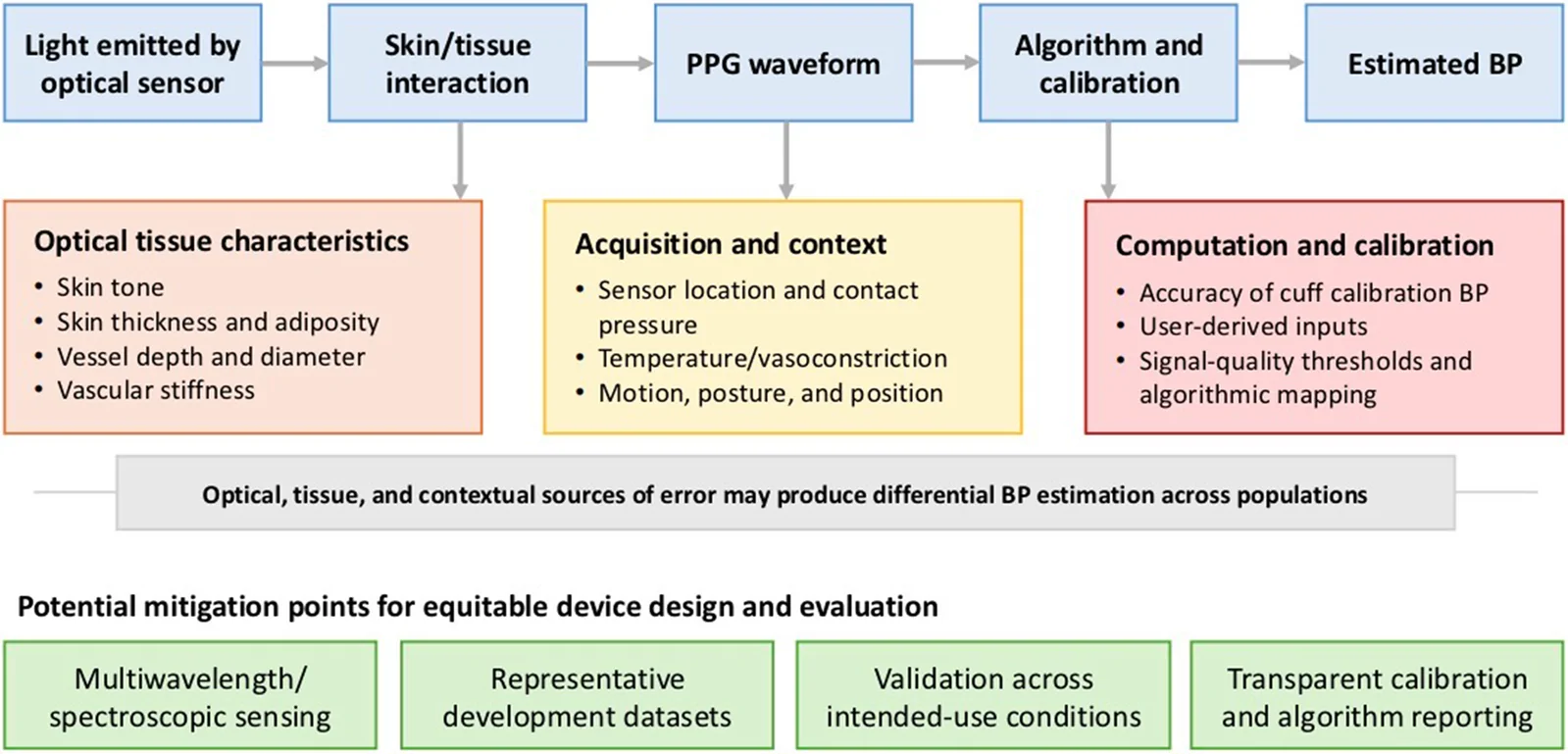
Potential sources of differential error in photoplethysmography-based cuffless BP measurement

However, cuffless devices estimate rather than directly measure BP. Most rely on photoplethysmography (PPG), tonometry, or other cardiac or pulsatility signals, with waveform features translated into BP estimates using mathematical or machine learning algorithms [38]. Many devices require initial and periodic calibration using cuff-based BP measurement. Consequently, their accuracy can depend on the accuracy of the calibration device and the user''s cuff-based measurement technique, potentially carrying existing measurement inequities into the cuffless estimate. PPG-based technologies raise additional equity considerations because signal quality and performance may be influenced by skin pigmentation, sensor location and contact pressure, ambient temperature, body habitus, vascular characteristics, movement, and body position Fig. 1 [40–43]. Vascular stiffness and tone are also relevant to cuffless approaches based on pulse transit or arrival time and waveform morphology, as these signals depend on vascular properties in addition to BP. Because vascular properties may differ across populations and change with antihypertensive treatment or other physiologic states, the relationship between the measured signal and BP may also change after calibration [38, 44]. Arterial stiffness has been reported to be higher on average among Black compared with White adults, although these differences are heterogeneous and partly reflect differences in BP and other cardiovascular risk factors [45]. Algorithms incorporating demographic or anthropometric inputs may introduce additional sources of bias when development and validation datasets do not adequately represent the populations in whom the devices will be used [46].

Validation and clinical interpretation of cuffless BP device output remain major limitations. Regulatory clearance does not necessarily indicate that a cuffless device has undergone formal clinical validation using a protocol appropriate for its technology and intended use [47]. Available validation approaches also do not consistently evaluate performance during real-world activities, sleep, changes in body position, antihypertensive treatment, over the full interval between calibrations, or across different skin tones [38]. Accordingly, current hypertension guidelines recommend against relying on cuffless devices for the diagnosis or management of hypertension until their precision and reliability improve [3]. Recent device-specific studies demonstrate substantial limitations under intended use conditions [48, 49], reinforcing that findings from one technology, population, or use condition cannot be generalized to cuffless BP monitoring as a whole. Notably, a 2026 independent evaluation of remote PPG cuffless BP technology in Nigeria found poor agreement with cuff-based BP and greater measurement failure with the darkest skin-tone category [50]. These results illustrate the importance of evaluating measurement accuracy across skin tones and conditions intended for real-world use.

From an equity perspective, cuffless BP monitoring represents both an opportunity and a potential source of new disparities. Future validation should deliberately include participants with diverse skin tones and populations varying by age, body habitus, pregnancy status, arrhythmia, vascular characteristics, and physical activity, while evaluating accuracy across the device''s intended use conditions and over time [38, 39]. Research must also address affordability, connectivity, digital literacy, data privacy, algorithm transparency, and whether the large volume of BP data generated by these devices improves clinical decisions and patient outcomes. Without attention to these factors, cuffless technologies may shift rather than eliminate inequities in BP measurement.

### Addressing Disparities and Moving Towards Equitable Blood Pressure Measurement

In order to address these disparities and move towards more equitable BP measurement, change is needed along several tiers including policy and healthcare systems, technologic innovations as well as patient centered strategies.

### Policy and Healthcare System Interventions

In 2019, the Centers for Medicare and Medicaid Services’ (CMS) revised its reimbursement of ABPM services for the first time 15 years and expanded its coverage from just suspected white coat hypertension to include suspected masked hypertension [51]. However, nationally specified coverage remains limited to these indications, despite broader clinical applications of ABPM [52]. Similarly, beginning in 2020, limited reimbursement pathways became available for SMBP device setup and patient training and for monthly BP data review and treatment-plan adjustment [53]. Further policy changes expanding insurance coverage of ABPM and HBPM is strongly needed to bridge the gap between its clinical applications and its current limited use.

Healthcare system changes are also needed to address disparities, which begins with standardizing BP measurement among healthcare providers. In a joint statement from thirteen scientific health organizations around the globe, Cheung et al. called for action emphasizing the importance of a pragmatic, standardized approach to clinic BP measurement [54]. Practice guidelines have delineated detailed instructions on optimal BP measurement, unfortunately, standardization is not implemented in most clinical practices [55]. Accordingly, Cheung et al. strongly recommended adopting streamlined protocols including several aspects of BP measurement [54]. The setting and equipment used must be standardized and include a clinically validated device with a range of cuff sizes available for use. Furthermore, healthcare professionals must be trained with annual retraining recommended. Patient preparation techniques and accurate measurement procedures should follow a standard protocol as well [54]. A systematic review of 328 articles identified 29 different components of BP measurement procedure that can affect the accuracy of the readings [13]. Without a standardized protocol, highly variable and inaccurate BP readings from one visit to the next are more likely, making achieving a specific BP goal extremely challenging.

Community based BP monitoring programs can also aid in reducing barriers to care. This was evident through the participation of Community Health Centers in the National Hypertension Control Initiative (NHCI) that introduced self-measured BP interventions to populations disproportionately impacted by hypertension including Black, Hispanic and American Indian/Alaskan Native persons. Participating programs experienced improvements in BP control from 44 to 69% at the SWLA Center for Health services in southwest Louisiana and from 53 to 70% at Canyonlands Healthcare in rural Arizona [56]. These experiences illustrate the potential for community health centers to implement team-based, culturally responsive approaches to improve BP control in populations experiencing barriers to care.

### Technological Innovations and Inclusive Device Development

Validated BP devices are more likely to be accurate compared to those that have not been validated [57]. However, most international regulatory bodies allow devices to be cleared for marketing and sale without sufficient evidence of independent, rigorous validation. In a study evaluating over 3411 devices from 457 different manufacturers that are available across the globe, only 20% were validated or reported by device companies to be equivalent to a validated device [58]. Currently, in order to ensure the validity of BP devices, the onus is on the consumer to check a validated device list [59]. An international initiative was taken on by the International Organization for Standardization, the United States Association for the Advancement of Medical Instrumentation, the European Society of Hypertension, the British Hypertension Society, and the Working Group on BP who agreed to develop a universal standard for device validation [38, 39, 60]. A call to action for regulatory bodies to require adherence to rigorous validation prior to clearance is essential to improve the accessibility of validated measurement devices. Of note, in the development of a single universal validation protocol, special populations were identified, including age, arm circumference, pregnancy, and atrial fibrillation, where there is theoretical and clinical evidence of the difference in the accuracy of BP monitors [60]. Current validation standards do not consistently require evaluation of device performance across skin tones or representative enrollment across racial and ethnic groups. Greater attention to representative enrollment and evaluation of device performance across diverse populations will be increasingly important as new BP measurement technologies are developed and implemented. Equitable device development should also consider whether devices can be used independently by individuals with disabilities. For example, recent evaluation of commercially available HBPM devices among individuals with blindness or low vision identified barriers related to inaccessible displays and instructions and limited tactile or audible guidance, highlighting the importance of accessibility alongside measurement accuracy [61].

### Patient-Centered Strategies

Patient centered strategies are also an integral component of addressing disparities in BP measurement particularly among groups experiencing inequities. This starts with adequate patient education to equip them with the competency needed for accurate BP self-measurement. Illustrating this, group-based hypertension self-management classes were offered in an urban Federally Qualified Health Center targeting particularly Black and Hispanic men [62]. The classes aimed to educate patients about hypertension, offer them skill-building measurement techniques and provided them with a validated BP cuff to use at home. Participants were trained in BP self-monitoring based on AHA and AMA joint recommendations. Among participants who attended multiple classes, most of whom were Black men, mean BP declined by 19.1/14.8 mmHg. As care is shifting to value-based models, expanding educational resources can create opportunities to reduce health disparity.

Patient involvement with HBPM, combined with clinical support, has been shown to be effective in improving BP control [63]. However, HBPM has several barriers that limit its use. These include patients’ concerns over the validity of home BP devices, the cost of the device, the reliability of BP measurement in a non-clinical setting and the absence of motivation to perform multiple readings [26]. A lack of overall knowledge about BP measurement procedures was also identified as patients have observed inconsistent measurement techniques while at physician’s offices which adds to their uncertainty and decreases their confidence in the accuracy of self-measurement. Some measurement-related barriers may be addressed through clear, technique-specific patient education. For example, the AHA Target: BP initiative provides illustrated, multilingual instructions for appropriate use of wrist BP monitors, including correct device positioning and maintenance of the wrist at heart level [34]. More broadly, the use of culturally and linguistically adapted hypertension education to address patients’ perceptions of hypertension has been shown to improve adherence to lifestyle recommendations and medications [64]. Similar training programs aimed at educating practice providers have also demonstrated positive learning experiences with adequate practice behavioral changes [65]. Increasing evidence is demonstrating that culturally adapted patient education combining culturally competent care with patient-centered care may better support underrepresented patients in chronic disease management such as hypertension [66–68].

### Research Priorities for Equitable BP Measurement

**Fig. 2:**
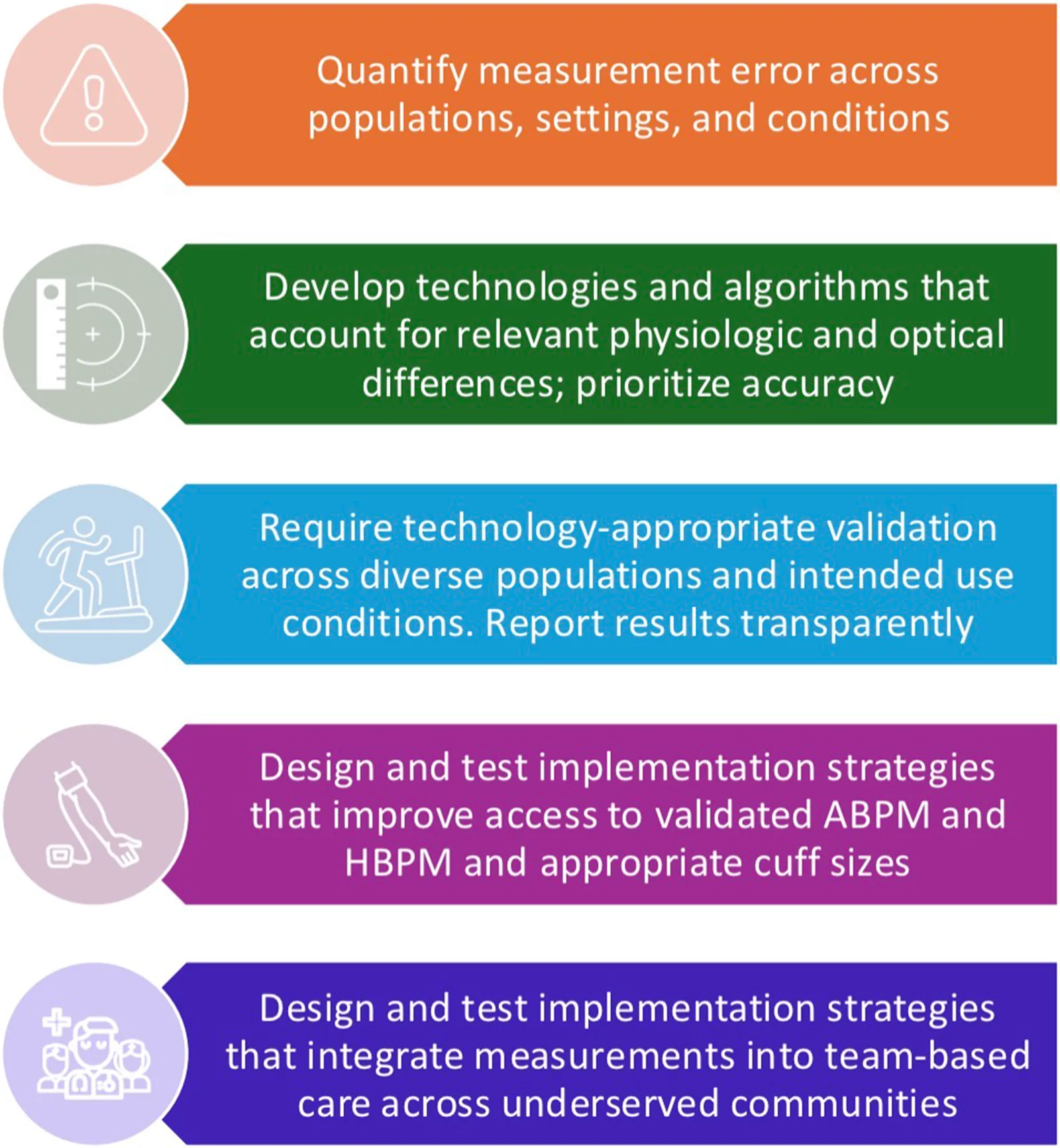
Research priorities to advance equitable blood pressure measurement

Future research should move beyond documenting differences in device performance across populations towards identifying and mitigating the mechanisms that produce these differences Fig. 2. For example, for optical technologies such as PPG, emerging approaches using multiwavelength optical sensing and direct characterization of skin optical properties may help distinguish physiologic signals from variation related to skin tone and other tissue characteristics [69]. Such approaches could inform both device design and more rigorous validation across skin tones. Importantly, these technologies will require evaluation specifically for BP estimation and under intended conditions of use before they can be assumed to reduce disparities in BP measurement. More broadly, research priorities should include quantifying measurement error across populations and settings; ensuring representative enrollment in device validation studies; identifying physiologic, technical, and contextual sources of differential measurement performance; evaluating novel approaches to mitigate these sources of error; testing implementation strategies to improve equitable access to ABPM and validated HBPM; and determining whether measurement-focused interventions ultimately reduce disparities in hypertension diagnosis, treatment, and cardiovascular outcomes.

## Conclusion

Disparities in hypertension diagnosis and control can be compounded by inequities in how BP is measured. Errors in OBPM technique and cuff sizing, limited access to ABPM and validated HBPM, and uneven implementation of standardized measurement can contribute to systematic misclassification among patients who already bear a disproportionate burden of hypertension. Emerging cuffless technologies may reduce some barriers but could introduce new sources of bias through calibration, skin-tone-related variation in optical sensor performance, algorithm design, and unequal access. Improving BP measurement equity requires attention both to the accuracy of individual devices and whether accurate measurement can be implemented consistently across populations and care settings. The next priority is to move beyond documenting differences in measurement performance and access toward testing interventions that reduce measurement bias and improve hypertension control in populations most affected by current gaps. Ultimately, equitable BP measurement should be treated as a prerequisite for equitable hypertension care rather than simply a technical component of it.

## Data Availability

No datasets were generated or analysed during the current study.

## References

[1] Muntner P, Shimbo D, Carey RM, Charleston JB, Gaillard T, Misra S, et al. Measurement of blood pressure in humans: a scientific statement From the American Heart Association. Hypertension. 2019;73(5):e35–66. 10.1161/HYP.0000000000000087 PMC11409525 30827125

[2] Zhou B, Perel P, Mensah GA, Ezzati M. Global epidemiology, health burden and effective interventions for elevated blood pressure and hypertension. Nat Rev Cardiol. 2021;18(11):785–802. 10.1038/s41569-021-00559-8 PMC8162166 34050340

[3] Jones DW, Ferdinand KC, Taler SJ, Johnson HM, Shimbo D, Abdalla M, et al. 2025 AHA/ACC/AANP/AAPA/ABC/ACCP/ACPM/AGS/AMA/ASPC/NMA/PCNA/SGIM guideline for the prevention, detection, evaluation and management of high blood pressure in adults: a report of the American College of Cardiology/American Heart Association Joint Committee on Clinical Practice Guidelines. Hypertension. 2025;82(10):e212–316. 10.1161/HYP.0000000000000249 40811516

[4] Fryar CD, Kit B, Carroll MD, Afful J. Hypertension prevalence, awareness, treatment, and control among adults age 18 and older: United States, August 2021–August 2023. NCHS Data Brief. 2024;511:1–10. 10.15620/cdc/164016 PMC13655856 40085792

[5] Foti K, Wang D, Appel LJ, Selvin E. Hypertension Awareness, Treatment, and Control in US Adults: Trends in the Hypertension Control Cascade by Population Subgroup (National Health and Nutrition Examination Survey, 1999–2016). Am J Epidemiol. 2019;188(12):2165–74. 10.1093/aje/kwz177 PMC7212399 31504121

[6] Ferdinand KC, Ali A, Echols MR. Racial/Ethnic considerations in the prevention of cardiovascular disease. In: Wong ND, Amsterdam EA, Toth PP, editors. ASPC Manual of Preventive Cardiology. Contemporary Cardiology. Springer, Cham. 2021; p. 463–87.

[7] Booth JN, Anstey DE, Bello NA, Jaeger BC, Pugliese DN, Thomas SJ, et al. Race and sex differences in asleep blood pressure: the Coronary Artery Risk Development in Young Adults (CARDIA) study. J Clin Hypertens (Greenwich). 2019;21(2):184–92. 10.1111/jch.13474 PMC6375074 30719843

[8] Pogue V, Rahman M, Lipkowitz M, Toto R, Miller E, Faulkner M, et al. Disparate estimates of hypertension control from ambulatory and clinic blood pressure measurements in hypertensive kidney disease. Hypertension. 2009;53(1):20–7. 10.1161/HYPERTENSIONAHA.108.115154 19047584

[9] Akinyelure OP, Jaeger BC, Oparil S, Carson AP, Safford MM, Howard G, et al. Social determinants of health and uncontrolled blood pressure in a national cohort of black and white US Adults: the REGARDS Study. Hypertension. 2023;80(7):1403–13. 10.1161/HYPERTENSIONAHA.122.20219 PMC10330022 37082942

[10] Abdalla M, Bolen SD, Brettler J, Egan BM, Ferdinand KC, Ford CD, et al. Implementation strategies to improve blood pressure control in the United States: a scientific statement from the American Heart Association and American Medical Association. Hypertension. 2023;80(10):e143–57. 10.1161/HYP.0000000000000232 PMC10578150 37650292

[11] Redmond N, Booth JN 3rd, Tanner RM, Diaz KM, Abdalla M, Sims M, et al. Prevalence of masked hypertension and its association with subclinical cardiovascular disease in African Americans: Results from the jackson heart study. J Am Heart Assoc. 2016;5(3):e002284. 10.1161/JAHA.115.002284 PMC4943234 27025968

[12] Parati G, Pengo MF, Avolio A, Azizi M, Bothe TL, Burnier M, et al. Nocturnal blood pressure: pathophysiology, measurement and clinical implications. Position paper of the European Society of Hypertension. J Hypertens. 2025;43(8):1296–318. 10.1097/HJH.0000000000004053 PMC12237141 40509714

[13] Kallioinen N, Hill A, Horswill MS, Ward HE, Watson MO. Sources of inaccuracy in the measurement of adult patients’ resting blood pressure in clinical settings: a systematic review. J Hypertens. 2017;35(3):421–41. 10.1097/HJH.0000000000001197 PMC5278896 27977471

[14] Stergiou GS, Palatini P, Parati G, O’Brien E, Januszewicz A, Lurbe E, et al. 2021 European Society of Hypertension practice guidelines for office and out-of-office blood pressure measurement. J Hypertens. 2021;39(7):1293–302. 10.1097/HJH.0000000000002843 33710173

[15] Ishigami J, Charleston J, Miller ER 3rd, Matsushita K, Appel LJ, Brady TM. Effects of cuff size on the accuracy of blood pressure readings: The Cuff(SZ) randomized crossover trial. JAMA Intern Med. 2023;183(10):1061–8. 10.1001/jamainternmed.2023.3264 PMC10407761 37548984

[16] Tu J, Chen H, Zeng Q, Chen L, Guo Y, Chen K. Trends in obesity prevalence among adults with hypertension in the United States, 2001 to 2023. Hypertension. 2025;82(3):498–508. 10.1161/HYPERTENSIONAHA.124.24123 39758027

[17] Palatini P, Asmar R. Cuff challenges in blood pressure measurement. J Clin Hypertens (Greenwich). 2018;20(7):1100–3. 10.1111/jch.13301 PMC8030978 30003699

[18] Diaz KM, Veerabhadrappa P, Brown MD, Whited MC, Dubbert PM, Hickson DA. Prevalence, determinants, and clinical significance of masked hypertension in a population-based sample of African Americans: The Jackson Heart Study. Am J Hypertens. 2015;28(7):900–8. 10.1093/ajh/hpu241 PMC4481565 25499058

[19] Jaeger BC, Booth JN 3rd, Butler M, Edwards LJ, Lewis CE, Lloyd-Jones DM, et al. Development of predictive equations for nocturnal hypertension and nondipping systolic blood pressure. J Am Heart Assoc. 2020;9(2):e013696. 10.1161/JAHA.119.013696 PMC7033845 31914878

[20] Whelton PK, Carey RM, Aronow WS, Casey DE, Collins KJ, Dennison Himmelfarb C, et al. 2017 ACC/AHA/AAPA/ABC/ACPM/AGS/APhA/ASH/ASPC/NMA/PCNA Guideline for the prevention, detection, evaluation, and management of high blood pressure in adults: A report of the American College of Cardiology/American Heart Association Task Force on Clinical Practice Guidelines. Hypertension. 2018;71(6):e13–115. 10.1161/HYP.0000000000000065 29133356

[21] Viera AJ, Yano Y, Lin F-C, Simel DL, Yun J, Dave G, et al. Does this adult patient have hypertension?: The rational clinical examination systematic review. JAMA. 2021;326(4):339–47. 10.1001/jama.2021.4533 34313682

[22] Guirguis-Blake JM, Evans CV, Webber EM, Coppola EL, Perdue LA, Weyrich MS. Screening for hypertension in adults: Updated evidence report and systematic review for the US preventive services task force. JAMA. 2021;325(16):1657–69. 10.1001/jama.2020.21669 PMC13344483 33904862

[23] Demko J, Cohen JB. Ambulatory blood pressure monitoring. JAMA Intern Med. 2026;186(6):760–1. 10.1001/jamainternmed.2026.0118 41870465

[24] Kronish IM, Kent S, Moise N, Shimbo D, Safford MM, Kynerd RE, et al. Barriers to conducting ambulatory and home blood pressure monitoring during hypertension screening in the United States. J Am Soc Hypertens. 2017;11(9):573–80. 10.1016/j.jash.2017.06.012 PMC5595651 28734798

[25] Shimbo D, Kent ST, Diaz KM, Huang L, Viera AJ, Kilgore M, et al. The use of ambulatory blood pressure monitoring among Medicare beneficiaries in 2007–2010. J Am Soc Hypertens. 2014;8(12):891–7. 10.1016/j.jash.2014.09.015 PMC4262763 25492832

[26] Carter EJ, Moise N, Alcantara C, Sullivan AM, Kronish IM. Patient barriers and facilitators to ambulatory and home blood pressure monitoring: a qualitative study. Am J Hypertens. 2018;31(8):919–27. 10.1093/ajh/hpy062 PMC7190918 29788130

[27] Cepeda M, Pham P, Shimbo D. Status of ambulatory blood pressure monitoring and home blood pressure monitoring for the diagnosis and management of hypertension in the US: an up-to-date review. Hypertens Res. 2023;46(3):620–9. 10.1038/s41440-022-01137-2 PMC9813901 36604475

[28] Shimbo D, Artinian NT, Basile JN, Krakoff LR, Margolis KL, Rakotz MK, et al. Self-measured blood pressure monitoring at home: A joint policy statement from the American Heart Association and American Medical Association. Circulation. 2020;142(4):e42–63. 10.1161/CIR.0000000000000803 32567342

[29] McEvoy JW, McCarthy CP, Bruno RM, Brouwers S, Canavan MD, Ceconi C, et al. 2024 ESC Guidelines for the management of elevated blood pressure and hypertension. Eur Heart J. 2024;45(38):3912–4018. 10.1093/eurheartj/ehae178 39210715

[30] Mancia G, Kreutz R, Brunstrom M, Burnier M, Grassi G, Januszewicz A, et al. 2023 ESH Guidelines for the management of arterial hypertension The Task Force for the management of arterial hypertension of the European Society of Hypertension: Endorsed by the International Society of Hypertension (ISH) and the European Renal Association (ERA). J Hypertens. 2023;41(12):1874–2071. 10.1097/HJH.0000000000003480 37345492

[31] Cohen JB, Abdalla M, Jones DW, Shimbo D. Home blood pressure monitoring: getting it right this time. J Am Coll Cardiol. 2025;86(18):1476–8. 10.1016/j.jacc.2025.08.007 40900052

[32] Kaur E, Rayani A, Brady TM, Matsushita K. Arm size coverage of popular over-the-counter blood pressure devices and implications in US adults. Hypertension. 2024;81(10):e125–7. 10.1161/HYPERTENSIONAHA.124.23473 39236150

[33] Jackson SL, Gillespie C, Shimbo D, Rakotz M, Wall HK. Blood pressure cuff sizes for adults in the United States: National Health and Nutrition Examination Survey, 2015-2020. Am J Hypertens. 2015;35(11):923–8. 10.1093/ajh/hpac104 PMC10851131 36066190

[34] Target:BP. Using a Wrist Cuff to Measure Blood Pressure. https://targetbp.org/tools_downloads/using-a-wrist-cuff-to-measure-blood-pressure/. Updated 27 Jun 2025. Access 6 Sep 2026.

[35] Casiglia E, Tikhonoff V, Albertini F, Palatini P. Poor reliability of wrist blood pressure self-measurement at home: A population-based study. Hypertension. 2016;68(4):896–903. 10.1161/HYPERTENSIONAHA.116.07961 27550911

[36] Clark D 3rd, Woods J, Zhang Y, Chandra S, Summers RL, Jones DW. Home blood pressure telemonitoring with remote hypertension management in a rural and low-income population. Hypertension. 2021;78(6):1927–9. 10.1161/HYPERTENSIONAHA.121.18153 PMC8589114 34757773

[37] Ford CD, Cameron NA, Clark D 3rd, Derington CG, Hardy ST, Mattei J, et al. Rural health and health disparities in hypertension management: a scientific statement from the American Heart Association. Hypertension. 2026;83(8):e00263. 10.1161/HYP.0000000000000263 42345114

[38] Cohen JB, Byfield RL, Hardy ST, Juraschek SP, Houston Miller N, Mukkamala R, et al. Cuffless devices for the measurement of blood pressure: A scientific statement from the American Heart Association. Hypertension. 2026;83(3):e00254. 10.1161/HYP.0000000000000254 PMC13335599 41376592

[39] Stergiou GS, Avolio AP, Palatini P, Kyriakoulis KG, Schutte AE, Mieke S, et al. European Society of Hypertension recommendations for the validation of cuffless blood pressure measuring devices: European Society of Hypertension Working Group on Blood Pressure Monitoring and Cardiovascular Variability. J Hypertens. 2023;41(12):2074–87. 10.1097/HJH.0000000000003483 37303198

[40] Keller MD, Harrison-Smith B, Patil C, Arefin MS. Skin colour affects the accuracy of medical oxygen sensors. Nature. 2022;610(7932):449–51. 10.1038/d41586-022-03161-1 36261563

[41] Ferryman K, Crews DC, Drabo EF, Iwashyna TJ, Kane O, Jackson JW. Adherence to FDA guidance on pulse oximetry testing among diverse individuals, 1996-2024. JAMA. 1996;333(7):631–2. 10.1001/jama.2024.26473 PMC11836753 39786431

[42] Fine J, Branan KL, Rodriguez AJ, Boonya-Ananta T, Ajmal, Ramella-Roman JC, et al. Sources of inaccuracy in photoplethysmography for continuous cardiovascular monitoring. Biosens (Basel). 2021;11(4):126. 10.3390/bios11040126 PMC8073123 33923469

[43] Chandrasekhar A, Yavarimanesh M, Natarajan K, Hahn JO, Mukkamala R. PPG sensor contact pressure should be taken into account for cuff-less blood pressure measurement. IEEE Trans Biomed Eng. 2020;67(11):3134–40. 10.1109/TBME.2020.2976989 PMC8856571 32142414

[44] McNally RJ, Boguslavskyi A, Malek R, Floyd CN, Cecelja M, Douiri A, et al. Influence of blood pressure reduction on pulse wave velocity in primary hypertension: a meta-analysis and comparison with an acute modulation of transmural pressure. Hypertension. 2024;81(7):1619–27. 10.1161/HYPERTENSIONAHA.123.22436 PMC11177599 38721709

[45] Buie JNJ, Stanley A, Nietert PJ, Logan A, Adams RJ, Magwood GS. Racial disparities in arterial stiffness between healthy whites and African Americans in the United States: a meta-analysis. J Natl Med Assoc. 2019;111(1):7–17. 10.1016/j.jnma.2018.06.001 PMC6620781 30129482

[46] Siddique SM, Tipton K, Leas B, Jepson C, Aysola J, Cohen JB, et al. The impact of health care algorithms on racial and ethnic disparities : A systematic review. Ann Intern Med. 2024;177(4):484–96. 10.7326/M23-2960 38467001

[47] Cohen JB, Brady TM, Juraschek SP, Picone DS, Yang E, Schutte AE. Apple Watch for hypertension screening. Hypertension. 2026;83(2):e26031. 10.1161/HYPERTENSIONAHA.125.26031 PMC12826266 41564145

[48] Tan I, Gnanenthiran SR, Chan J, Kyriakoulis KG, Schlaich MP, Rodgers A, et al. Evaluation of the ability of a commercially available cuffless wearable device to track blood pressure changes. J Hypertens. 2023;41(6):1003–10. 10.1097/HJH.0000000000003428 PMC10158604 37016925

[49] Almeida TP, Perruchoud D, Alexandre J, Vermare P, Sola J, Shah J, et al. Evaluation of Aktiia cuffless blood pressure monitor across 24-h, daytime, and night-time measurements versus ambulatory monitoring: a prospective, single-centre observational study. J Hypertens. 2025;43(4):690–7. 10.1097/HJH.0000000000003960 PMC11872257 39927495

[50] Dasa D, Davies P. Skin tone and diagnostic equity in contactless blood pressure screening: a prospective observational field evaluation of remote photoplethysmography in Nigeria. BMJ Open. 2026;16(6):e119311. 10.1136/bmjopen-2026-119311 PMC13288978 42331583

[51] Decision Memo for Ambulatory Blood Pressure Monitoring (ABPM) (CAG-00067R2). https://www.cms.gov/medicare-coverage-database/details/nca-decision-memo.aspx?NCAId=294. Accessed 1 Jan, 2026. 2019.

[52] Dixon DL, Salgado TM, Luther JM, Byrd JB. Medicare reimbursement policy for ambulatory blood pressure monitoring: a qualitative analysis of public comments to the Centers for Medicare and Medicaid Services. J Clin Hypertens (Greenwich). 2019;21(12):1803–9. 10.1111/jch.13719 PMC7018518 31642596

[53] Tang M, Nakamoto CH, Stern AD, Zubizarreta JR, Marcondes FO, Uscher-Pines L, et al. Effects of remote patient monitoring use on care outcomes among medicare patients with hypertension an observational study. Ann Intern Med. 2023;176(11):1465–75. 10.7326/M23-1182 PMC13400060 37931262

[54] Cheung AK, Whelton PK, Muntner P, Schutte AE, Moran AE, Williams B, et al. International consensus on standardized clinic blood pressure measurement - a call to action. Am J Med. 2023;136(5):438-45 e1. 10.1016/j.amjmed.2022.12.015 PMC10159895 36621637

[55] Stergiou G, Kollias A, Parati G, O’Brien E. Office blood pressure measurement: the weak cornerstone of hypertension diagnosis. Hypertension. 2018;71(5):813–5. 10.1161/HYPERTENSIONAHA.118.10850 29531176

[56] Smith A, Wagner A, Brown L, Cardoso C, Dadkhah M, Decuir C, et al. Abstract 090: Using evidence-based resources and unique self-measured blood pressure program designs to improve blood pressure control. Hypertension. 2023;80(Suppl_1). 10.1161/hyp.80.suppl_1.090.

[57] Akpolat T, Aydogdu T, Erdem E, Karatas A. Inaccuracy of home sphygmomanometers: a perspective from clinical practice. Blood Press Monit. 2011;16(4):168–71. 10.1097/mbp.0b013e328348ca52 21928543

[58] Picone DS, Campbell NRC, Schutte AE, Olsen MH, Ordunez P, Whelton PK, et al. Validation status of blood pressure measuring devices sold globally. JAMA. 2022;327(7):680–1. 10.1001/jama.2021.24464 PMC8848194 35166811

[59] Picone DS, Padwal R, Campbell NRC, Boutouyrie P, Brady TM, Olsen MH, et al. How to check whether a blood pressure monitor has been properly validated for accuracy. J Clin Hypertens (Greenwich). 2020;22(12):2167–74. 10.1111/jch.14065 PMC8030032 33017506

[60] Stergiou GS, Alpert B, Mieke S, Asmar R, Atkins N, Eckert S, et al. A universal standard for the validation of blood pressure measuring devices: Association for the Advancement of Medical Instrumentation/European Society of Hypertension/International Organization for Standardization (AAMI/ESH/ISO) Collaboration Statement. J Hypertens. 2018;36(3):472–8. 10.1097/HJH.0000000000001634 PMC5796427 29384983

[61] Hernandez-Sanchez D, Kazantceva A, Nizam A, Salehi Z, Knot M, Watkins AR, et al. The path towards an accessible blood pressure monitor for persons living with low vision or blindness: a Canadian pilot study. Disabil Rehabil Assist Technol. 2026;21(6):3273–83. 10.1080/17483107.2026.2635508 41774054

[62] Eck C, Biola H, Hayes T, Bulgin D, Whitney C, Raman R, et al. Efficacy of hypertension self-management classes among patients at a federally qualified health center. Prev Chronic Dis. 2021;18:E70. 10.5888/pcd18.200628 PMC8300538 34264812

[63] Uhlig K, Patel K, Ip S, Kitsios GD, Balk EM. Self-measured blood pressure monitoring in the management of hypertension: a systematic review and meta-analysis. Ann Intern Med. 2013;159(3):185–94. 10.7326/0003-4819-159-3-201308060-00008 23922064

[64] Beune EJ, van Moll EP, Beem L, Mohrs J, Agyemang CO, Ogedegbe G, et al. Culturally adapted hypertension education (CAHE) to improve blood pressure control and treatment adherence in patients of African origin with uncontrolled hypertension: cluster-randomized trial. PLoS ONE. 2014;9(3):e90103. 10.1371/journal.pone.0090103 PMC3943841 24598584

[65] Meinema JG, Haafkens JA, Jaarsma D, van Weert H, van Dijk N. Development and evaluation of a culturally appropriate hypertension education (CAHE) training program for health care providers. PLoS ONE. 2017;12(6):e0178468. 10.1371/journal.pone.0178468 PMC5464541 28594878

[66] Netto G, Bhopal R, Lederle N, Khatoon J, Jackson A. How can health promotion interventions be adapted for minority ethnic communities? Five principles for guiding the development of behavioural interventions. Health Promot Int. 2010;25(2):248–57. 10.1093/heapro/daq012 20299500

[67] Saha S, Beach MC, Cooper LA. Patient centeredness, cultural competence and healthcare quality. J Natl Med Assoc. 2008;100(11):1275–85. 10.1016/s0027-9684(15)31505-4 PMC2824588 19024223

[68] Cooper LA, Hill MN, Powe NR. Designing and evaluating interventions to eliminate racial and ethnic disparities in health care. J Gen Intern Med. 2002;17(6):477–86. 10.1046/j.1525-1497.2002.10633.x PMC1495065 12133164

[69] Rathod M, Wu J, Unger S, Ross H, Franklin D. Characterizing effects of skin pigmentation and anatomical location in reflective photoplethysmography. SPIE. 2026;13848. 10.1117/12.3081535.

